# Label-Free Identification of Early Stages of Breast Ductal Carcinoma via Multiphoton Microscopy

**DOI:** 10.1155/2020/9670514

**Published:** 2020-04-02

**Authors:** Zhong Chen, Wenhui Guo, Deyong Kang, Shu Wang, Liqin Zheng, Gangqin Xi, Yuane Lian, Chuan Wang, Jianxin Chen

**Affiliations:** ^1^Key Laboratory of OptoElectronic Science and Technology for Medicine of Ministry of Education, Fujian Provincial Key Laboratory of Photonics Technology, Fujian Normal University, Fuzhou 350007, China; ^2^Department of Breast Surgery, Fujian Medical University Union Hospital, Fuzhou 350001, China; ^3^Department of Pathology, Fujian Medical University Union Hospital, Fuzhou 350001, China; ^4^College of Mechanical Engineering and Automation, Fuzhou University, Fuzhou 350116, China

## Abstract

Breast cancer can be cured by early diagnosis. Appropriate and effective clinical treatment benefits from accurate pathological diagnosis. However, due to the lack of effective screening and diagnostic imaging methods, early stages of breast cancer often progress to malignant breast cancer. In this study, multiphoton microscopy (MPM) via two-photon excited fluorescence combined with second-harmonic generation was used for identifying the early stages of breast ductal carcinoma. The results showed differences in both cytological features and collagen distribution among normal breast tissue, atypical ductal hyperplasia, low-grade ductal carcinoma in situ, and high-grade ductal carcinoma in situ with microinvasion. Furthermore, three features extracted from the MPM images were used to describe differences in cytological features, collagen density, and basement membrane circumference in the early stages of breast ductal carcinoma. They revealed that MPM has the ability to identify early stages of breast ductal carcinoma label-free, which would contribute to the early diagnosis and treatment of breast cancer. This study may provide the groundwork for the further application of MPM in the clinic.

## 1. Introduction

Breast cancer is the most frequent cancer in women and the second most common cancer causing female death, with estimated 266,120 new cases and 40,920 deaths in 2018 in the USA [1]. However, 70%-75% of breast cancers are associated with breast ductal lesions [2]. Atypical ductal hyperplasia (ADH) is an indirect precursor of invasive ductal carcinoma, and ductal carcinoma in situ (DCIS) is an immediate precursor of invasive ductal carcinoma [3–6]. Ductal carcinoma in situ with microinvasion (DCIS-MI) is a prestage of breast ductal carcinoma [7, 8]. If these early lesions are detected in time, diagnosed accurately, and treated early, patients will have a good prognosis. DCIS is a group of highly heterogeneous neoplastic intraductal lesions that classify into low-grade DCIS, intermediate-grade DCIS, and high-grade DCIS, primarily based on the nuclear grade or necrosis [9]. Atypical hyperplasia of ADH is similar to proliferating cells of low-grade DCIS, but the extent of ADH is small, with less than two lumens completely involved, or lesions less than 2 mm [10, 11]. DCIS-MI is characterized by ductal carcinoma in situ, while some cancer cells break through the basement membrane of the duct system into adjacent stromal tissue with no foci more than 1 mm of the maximum diameter [8]. DCIS-MI often occurs in high-grade DCIS with a wide range of lesions but can also be seen in any grade of DCIS [7, 8]. However, due to the lack of accurate and rapid screening as well as diagnostic imaging methods, these lesions often progress to malignant ductal carcinoma and even distant metastases.

Currently, pathologists identify these early stages of breast ductal carcinoma through hematoxylin and eosin- (H&E-) stained sections of needle biopsy specimens or postoperative specimens. Although ADH and DCIS are easily identified by H&E sections, the identification of DCIS-MI is difficult. Pathologists identify acute microinvasive foci and other suspected lesions by using immunostaining markers to label myoepithelial cells around the tumor and seeing the presence of myoepithelial cell layers. However, when reslicing to detect immunostaining markers of infiltrates, microinvasive lesions may no longer appear on the sections. In addition, histopathological examination of H&E sections has several other shortcomings, including time-consuming and labor-intensive pathological processes and potential risk bias. Therefore, there is an urgent need for developing fast and efficient optical imaging methods that can be used for screening and diagnosing these early lesions.

Multiphoton microscopy (MPM) via two-photon excited fluorescence (TPEF) combined with second-harmonic generation (SHG) is a promising, label-free, and high-resolution imaging technique for cancer research and diagnosis [12–15]. There are abundant autofluorescent substances in breast tissue, such as elastic fibers, NAD(P)H, and FAD, which can generate TPEF signals [16, 17]; asymmetric collagen such as type I collagen, type II collagen, type III collagen, and type V collagen is more likely to produce SHG signals [18]. Particularly, SHG imaging visualizes the microstructure of collagen fibers and elastin fibers in the extracellular matrix by detecting tissue intrinsic fluorescence [19]. On the other hand, TPEF imaging can directly observe changes in cellular morphology and organization [12]. High-contrast SHG and TPEF overlay images highlight collagen reorganization in the tumor-stromal interface, which is associated with cancer progression. Hence, in this study, we sought to examine whether MPM imaging can identify early stages of breast ductal carcinoma.

## 2. Materials and Methods

### 2.1. Sample Preparations

In this work, 16 cases of paraffin-embedded breast cancer samples, including 5 normal breast tissues, 3 ADH, 5 low-grade DCIS, and 3 high-grade DCIS-MI, were obtained from the pathological department of Fujian Union Hospital. The patients enrolled in the study were diagnosed at 21-75 years old. The Institutional Review Committee of Fujian Union Hospital (Fuzhou, China) approved of this study, and patients signed informed consent prior to participating in this study. Five consecutive sections of 5 *μ*m thickness were used in this study, and after paraffin section dewaxing, the middle slice was stained with H&E to further determine experimental results; the other four sections were used for multiphoton imaging.

### 2.2. MPM System

The MPM system used in this work has been described in detail before [20]. Briefly, a commercial laser scanning microscope (Zeiss LSM 880 META, Jena, Germany) equipped with an external automode-locked Ti:sapphire laser (140 fs, 80 MHz) was used for all MPM imaging. Unstained samples were excited by 810 nm light from the tunable Ti:sapphire laser (Chameleon Ultra, Coherent, Inc., Santa Clara, CA, USA). A 20x Plan-Apochromat objective (NA = 0.8, Zeiss, Jena, Germany) was used for getting backscattered fluorescence, which was separated by passing through a grating onto 32-channel GaAsP and PMT array detectors to obtain a TPEF signal and SHG signal, respectively. MPM images were obtained from two separate channels simultaneously: (1) the TPEF channel gathered 430 nm-695 nm fluorescence with the 32-channel GaAsP detectors (color-coded red); (2) the SHG channel gathered 389 nm-419 nm fluorescence with a PMT detector (color-coded green). The LSM software automatically spliced out an image array with a resolution of 1024 × 1024 pixels per frame to form a large-area image. All MPM images had a 12-bit pixel depth; the images were obtained at 0.77 *μ*s/pixel. The maximum scanning speed in the bidirectional scanning mode was 1.6 frames/sec (1024 × 1024 pixels).

### 2.3. Histologic Analysis

H&E-stained digital images (40x) were obtained from an optical microscope (Eclipse Ci-L, Nikon Instruments, Japan) with CCD (DS-Fi2, Nikon) and confirmed by two experienced pathologists (Deyong Kang and Yuane Lian), reducing subjective observational errors. Then, two researchers (Liqin Zheng and Zhong Chen) confirmed the result of the multiphoton image by comparing the multiphoton image with the H&E-stained digital image.

### 2.4. Statistical Analysis

To further describe the differences between cytological features and collagen features in normal breast tissue, ADH, low-grade DCIS, and high-grade DCIS-MI, three features were quantified from MPM images. The nuclear area of the epithelial cells within the ducts was used to describe differences in cytological features within the ducts, and collagen density was used to describe differences in collagen content around the ducts, and the basement membrane circumference was used to describe the extent of lesion involvement in the ducts. All statistical analyses were done using *IBM SPSS Statistics 21*. Statistical significance between each group was determined using the one-way ANOVA and was considered statistically significant when the *P* value was <0.05.

## 3. Result

### 3.1. Multiphoton Microscopic Imaging

**Figure 1:**
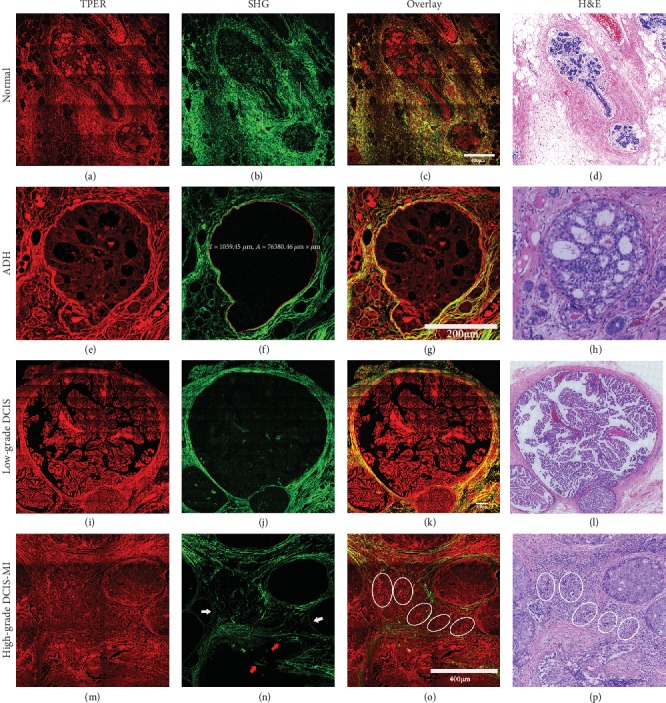
Representative MPM images and corresponding H&E images of normal breast tissue, ADH, low-grade DCIS, and high-grade DCIS-MI. White arrow: complete basement membrane; red arrow: broken basement membrane; white oval: microinvasion cancer cells.

**Figure 2:**
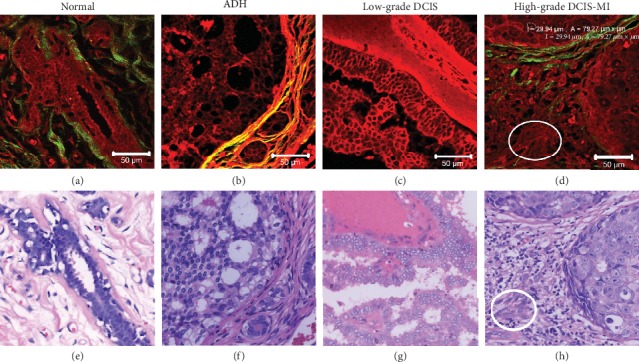
Representative MPM images and corresponding H&E images of the microstructure of normal breast tissue, ADH, low-grade DCIS, and high-grade DCIS-MI. White oval: microinvasion cancer cells; scale bar: 50 *μ*m.

To demonstrate whether MPM has the ability to reveal differences in microstructure between normal breast tissue, ADH, DCIS, and DCIS-MI, MPM images of different samples were obtained and compared with corresponding H&E images. During the multiphoton imaging scan of label-free breast tissue, a “screen door effect” appeared on the large-field multiphoton images (e.g., Figures 1(a), 1(b), 1(c), 1(i), 1(j), and 1(k)) causing a “grid” to appear on these images. However, when we divided a large-field-of-view image into four equal parts or eight equal parts for multiphoton imaging, the “screen door effect” was weakened significantly or does not appear on the multiphoton image.  Figure 1 shows the representative MPM images and corresponding H&E images of normal breast tissue, ADH, DCIS, and DCIS-MI.  Figure 2 shows representative MPM images and corresponding H&E images of the microstructure of normal breast tissue, ADH, low-grade DCIS, and high-grade DCIS-MI. As shown in Figure 1(c), normal breast tissue consisted of two parts: one was the ductal-lobular system and the other was the stromal system composed of fibrous tissue and adipose tissue. According to previous reports and current experimental results, cells and elastic fibers are capable of producing TPEF signals (red color-coded) in Figure 1(a), whereas collagen in the extracellular matrix produces SHG (red color-coded) in Figure 1(b) [1]. Specifically, Figure 2(a) showed that the normal ductal was bilayered: the inner cell layer consists of epithelial cells, while the outer layer contains myoepithelial cells. Collagen fibers within stromal tissue were curved and dense, and basement membranes of the acinus and duct can be seen in Figure 1(b). The cytological features and positional alignment information of these cells in the MPM images were consistent with the corresponding H&E images in Figures 1(d) and 2(e), whereas the H&E images would not directly observe the distribution of collagen and the presence of the basement membrane.

Unlike the normal duct, ADH was a small-scale proliferation of epithelial cells within the duct, and these epithelial cells were characterized by small uniform nuclei, as shown in the TPEF image (Figure 1(e)). As can be seen in Figure 1(f), the basement membrane of ADH excited strong SHG signals so that the basement membrane was clearly visible. Additionally, the MPM images (Figures 1(e)–1(g)) showed that the basement membrane was intact but significantly enlarged. The high-contrast overlay images (Figures 1(g) and 2(b)) clearly showed the distribution and microstructure of collagen fibers and proliferating epithelial cells. These morphological details correlate well with the results of H&E images (Figures 1(h) and 2(f)). However, H&E images failed to show the microstructure and distribution of collagen fibers directly.

Figures 1(i)–1(l) show an example of MPM images and a corresponding H&E image of low-grade DCIS.  Figure 2 shows that epithelial cells proliferating in low-grade DCIS ducts were similar to those proliferating in ADH ducts. Hyperplastic epithelial cell populations are characterized by small and uniform nuclei. Unlike ADH, low-grade DCIS involved more ducts or involved a larger duct (Figures 1(g) and 1(k)). As shown in Figure 1(k), epithelial cells proliferating in ducts involved a large duct and adjacent ducts. The strong SHG signal showed that the collagen fiber bundles in the basement membrane and stromal tissue were clearly visible, and the collagen fibers became straight due to the expansion of the ducts. The MPM images (Figure 2(c)) of low-grade DCIS showed details of the hyperplastic epithelial cells that showed exactly the same details as the H&E image (Figure 2(g)). However, the SHG image (Figure 1(j)) showed the distribution of collagen in the stromal tissue, which could not be directly observed from the H&E images.

Compared with the ADH and DCIS, DCIS-MI is based on DCIS with cancer cells breaking through the basement membrane of the duct into the stromal tissue, but the maximum diameter of the largest infiltrating lesion is ≤1 mm. Figures 1(m)–1(p) show MPM images of high-grade DCIS-MI and a corresponding H&E-stained image of high-grade DCIS-MI. Unlike the proliferating cells of low-grade DCIS, the proliferating cells of high-grade DCIS had larger cell volume, larger nuclei, obvious nuclear abnormalities, and nucleoli (Figure 1(m)). The SHG image (Figure 1(n)) displayed that the collagen around the ducts was unevenly distributed, and some of the collagen around the ducts was very thin, while some of the collagen around the ducts was very dense. The basement membrane of one of the ducts was destroyed, the collagen in the stromal tissue was no longer distributed around the duct but disorderly arranged, and the collagen density was also reduced as shown in Figure 1(n). The cytological features exhibited by MPM images were identical to those shown by H&E images (Figures 1(p) and 2(h)), but the H&E images were not able to directly exhibit morphological changes in the collagen in the basement membrane and stromal tissue.

Comparing MPM images of normal breast tissue and early stages of breast ductal carcinoma, including ADH, low-grade DCIS, and high-grade DCIS-MI, MPM-identifiable features are summarized and shown in Table 1. MPM had the ability to identify early stages of breast ductal carcinoma by combining TPEF signals from cells and SHG signals from collagen in the stromal tissue. It revealed that MPM had the ability to provide accurate pathology information for clinical diagnosis.

### 3.2. Quantitative Analysis

**Figure 3:**
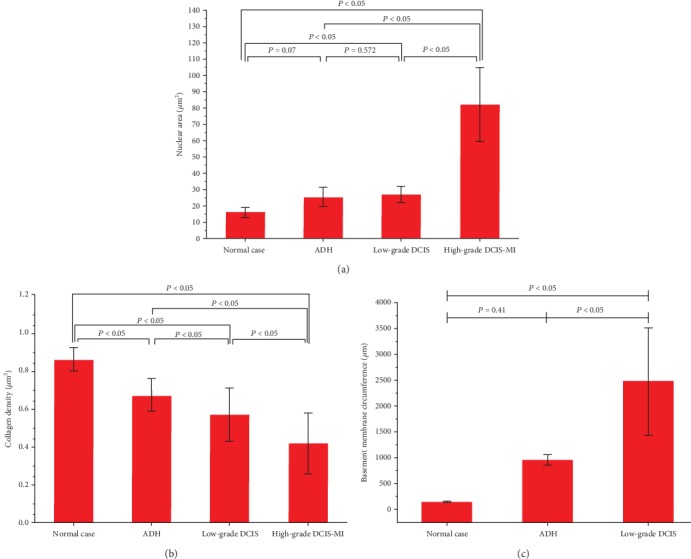
Nuclear area (a) and collagen density (b) of normal breast tissue versus ADH and low-grade DCIS as well as high-grade DCIS-MI; the basement membrane circumference (c) of normal breast tissue versus ADH and low-grade DCIS; error bars: standard deviation.

In Figure 2, the TPEF signal intensity varies greatly between the nucleus and the cytoplasm, so we can manually draw the outline of the nucleus. As shown in Figure 2(d), we automatically got the nucleus area after manually drawing the outline of the nucleus in the ZEN back software. The nucleus was circled out manually on the ZEN back software to measure the size of the nucleus. Twenty well-defined nuclei were selected randomly from each normal, AHD, low-grade DCIS, and high-grade DCIS-MI sample. Specifically, the nuclear area was 16.12 ± 3.10 *μ*m^2^ in normal breast tissue, 25.75 ± 5.62 *μ*m^2^ in ADH, 27.10 ± 5.28 *μ*m^2^ in low-grade DCIS, and 82.25 ± 22.76 *μ*m^2^ in high-grade DCIS-MI. According to the one-way ANOVA test, we provided a bar chart (Figure 3(a)) of the statistics to show the significance between the different groups. Our results showed no statistical nuclear area difference (*P* > 0.05) between normal breast tissue and ADH (*P* = 0.07) and between ADH and low-grade DCIS (*P* = 0.572). However, there were statistically significant differences between the other two groups (*P* < 0.05).

For each sample, three random SHG images of the same size (512 × 512 pixels) were selected to calculate the collagen density. Collagen density is defined as the ratio of the pixel number A to the total of B, and the pixel number A is the pixel number of the total pixel number B minus the low threshold number (low-intensity SHG signal and background signal). To avoid the influence of the “grid” on the quantitative measurement of collagen density, we chose the measurement position (ROI) in the middle of each “grid (size 1024 × 1024 pixels)”; for example, we selected the position and size of the white rectangle in Figure 1(b) to quantify the collagen density. The mean and SD of the collagen density were 0.86 ± 0.06 for normal breast tissue, 0.67 ± 0.09 for ADH, 0.57 ± 0.137 for low-grade DCIS, and 0.42 ± 0.162 for high-grade DCIS-MI. The bar chart (Figure 3(b)) of the statistics showed statistically significant difference (*P* < 0.05) in collagen density between any two groups.

To quantify the size of normal ducts and the size of lesions involved, we used the ZEN back software to manually circle the basement membrane and obtain automatically its circumference, as shown in Figure 1(f). To reduce the influence of the “grid” phenomenon on the quantitative measurement of the basement membrane circumference, we chose the duct basement membrane in the image without the “grid” for measurement, except for Figure 1(j). For normal and low-grade DCIS samples, three complete ducts were selected for each sample to calculate the basement membrane circumference. However, for ADH samples, one or two complete ducts were selected for each sample to calculate the basement membrane circumference. To be specific, the basement membrane circumference in normal ducts was 149.32 ± 12.56 *μ*m, in ADH ducts was 958.96 ± 101.63 *μ*m, and in low-grade DCIS was 2474.35 ± 1050.11 *μ*m, whereas, in high-grade DCIS-MI, it is “∞” because the high-grade DCIS-MI has some incomplete basement membranes. We did the one-way ANOVA test of the basement membrane circumference and found that there was no statistically significant difference (*P* > 0.05) between the normal case and ADH (*P* = 0.41), and the other two groups showed statistically significant difference (*P* < 0.05), as shown in Figure 3(c).

## 4. Discussion

MPM, targeting SHG and TPEF, has been widely used as a very useful tool in cancer research and detection, such as intraoperative real-time detection, marginal implementation assessment, and cancer diagnosis [13, 19, 21–24]. Moreover, MPM also plays an important role in breast cancer research, including rapid intraoperative evaluation of breast lesions, detection of breast tumor progression, and the role of collagen density in the mammary tumor initiation and progression [15, 25, 26]. However, few MPM studies have been conducted on these early stages of breast ductal carcinoma [16], especially on MPM imaging of ADH and DCIS-MI. In recent years, these early stages of breast ductal carcinoma have attracted more and more scholars' attention because of the good prognosis of these lesions after treatment [27]. The identification of DCIS and DCIS-MI is a difficult point in the breast pathological diagnosis. The presence of cancer cell infiltrates is a central concern for clinicians and pathologists because of the different treatment strategies for DCIS and DCIS-MI.

In the early-stage lesions of breast ductal carcinoma, there must be differences in cytological features, which are a very important indicator of tumor staging [25]. The nucleus of high-grade DCIS-MI was significantly larger than that of DCIS and ADH, as shown in Figures 2 and 3(a). The nuclei of normal breast tissue, ADH, and low-grade DCIS were mostly oval. However, the nuclei of high-grade DCIS-MI vary widely in size, with round and oval shapes. Additionally, breast lesions also have different changes in the extracellular matrix at different stages. We did not find any statistically significant differences in the nuclear area and basement membrane circumference between normal breast tissue and ADH (Figures 3(a) and 3(c)). But we found statistically significant differences in collagen density in the stromal tissue between normal breast tissue and ADH. Therefore, the ability to visualize cytological features and collagen morphology in the extracellular matrix is very helpful in identifying early stages of breast ductal carcinoma. In this study, we used MPM to detect cytological features and collagen structure characteristics by combining TPEF signals from intracellular autofluorescence and SHG signals from collagen in the extracellular matrix [28].

Our results reveal that MPM is an efficient and promising clinical tool for identifying early stages of breast ductal carcinoma. It not only provides high-resolution cytological features comparable to H&E-stained slice images of the “gold standard” of pathology but also provides additional collagen imaging information for extracellular matrices [22]. Quantitative results of nuclear area and basement membrane circumference revealed that ADH and low-grade DCIS have similar cytological features, but low-grade DCIS had a wider range of lesions, and involve more ducts. Compared with ADH and low-grade DCIS, high-grade DCIS-MI has larger nuclei, and some of the ductal basement membranes were destroyed, causing some cancer cells to propagate into adjacent stromal tissue. The quantitative statistical results of collagen density reflect changes of the extracellular matrix collagen content, which is an important indicator of tumor staging [25]. Therefore, these three variable analyses would enable identifying the early stages of breast ductal carcinoma.

Compared to fresh unfixed tissue samples, MPM imaging of paraffin section samples has a strong fluorescent background due to formalin fixation [29, 30]. Therefore, the image background is more uniform and can produce good image contrast with the nucleus or other components. In this study, TPEF signals were mainly generated from elastic fibers, NAD(P)H, FAD, and formalin solutions. The TPEF signal in the fixed high-grade nucleus mainly comes from the formalin solution and does not change with the change of fixed conditions. In Figures 1(o) and 2(d), we observed that there were one or more red dots (nucleoli) in the high-grade nuclei, but there was no TPEF signal (red dots) in the high-grade nuclei in fresh unfixed breast samples [25]. On the other hand, MPM imaging of fresh unfixed tissue samples can reduce the time for rapid intraoperative diagnosis because fresh samples do not require steps such as fixation and staining. However, in actual clinical procedures, fresh tissue samples of ADH and DCIS-MI are more difficult to collect.

With the increasing attention on MPM, many studies have demonstrated that MPM has great advantages in the feasibility of clinical translation [20, 25, 31, 32]. However, compared to currently accepted clinical imaging systems (ultrasound, X-ray computed tomography or tomosynthesis, and MRI), MPM has limited penetration depth for high-resolution imaging of tissues. Miniature multiphoton endoscopes and flexible small-diameter MPM probes overcome the previous limitations of this technology [33–36]. Huland et al. and Demirhan et al. have developed a compact and flexible multiphoton microendoscope to observe the liver, kidney, and colon of anesthetized rats [33, 34]. Yan et al. used a fluorescence endoscopy imaging system based on gradient index (GRIN) lenses to achieve *in vivo* dynamic fluorescence microendoscopic imaging and monitored the movement of subcutaneous blood flow in anesthetized mice [35]. In addition, Karsten et al. successfully exceeded the 600 Hz frame rate with a multifocal multiphoton endoscope [37]. Therefore, our studies on *ex vivo* human breast tissue will provide the groundwork for further application of MPM in vivo and in clinic, such as real-time diagnosis of intraoperative tissue and postoperative pathological analysis.

## 5. Conclusion

In conclusion, MPM is a very useful tool for label-free identification of early stages of breast ductal carcinoma, including ADH, low-grade DCIS, and high-grade DCIS-MI. Through high-contrast MPM images, we can analyze quantitatively the microstructural changes in the nucleus, basement membrane, and collagen density to distinguish the early stages of breast ductal carcinoma. Multiphoton microscopy via TPEF combined with SHG has the potential to be used for clinical diagnosis and lead to better management of these diseases.

## Figures and Tables

**Table 1:** MPM identifiable features of normal breast tissue, ADH, low-grade DCIS, and high-grade DCIS-MI.

Breast tissue sample	TPEF signal	SHG signal
Normal	Two layers of epithelial cells line in the duct	Curly and rich collagen fibers in the stromal Basement membrane outline is clearly visible and complete

ADH	Small-scale epithelial cells proliferate in the duct	The basement membrane remains intact but significantly enlarged Collagen around the basement membrane becomes fine as the duct expands No significant change in the morphology of the collagen in the stromal, but the collagen density is reduced
	Complete involvement of fewer than 2 ducts or <2 mm in extent	
Low-grade DCIS	Small, uniform cells with generally rounded nuclei that are evenly spaced cells, proliferate in the ducts	
	A large number of proliferating cells involve more than two ducts or involve large ducts >2 mm in diameter	

High-grade DCIS-MI	Large cells with distinct nucleoli, significant nuclear abnormalities, proliferate in the duct and stromal	Some basement membranes remain intact, but some basement membranes are no longer intact The collagen bundles in the stromal become straight and sparse
	A large amount of lymphocyte infiltration and a small amount of malignant cell infiltrate in the stroma	

## Data Availability

All data needed to evaluate the conclusions in the paper are present in the paper. Additional data related to this paper may be requested from the authors.
